# Rethinking mechanical heart valves in the aortic position: new paradigms in design and testing

**DOI:** 10.3389/fcvm.2024.1458809

**Published:** 2025-01-30

**Authors:** Sreyashi Chakraborty, Melinda G. Simon, Alessandro Bellofiore

**Affiliations:** Biomedical Engineering Department, San José State University, San Jose, CA, United States

**Keywords:** mechanical heart valves, thrombogenicity, aortic valve replacement, hemodynamics, particle image velocimetry

## Abstract

Bileaflet mechanical heart valves (MHV) remain a viable option for aortic valve replacement, particularly for younger patients and patients from low- and middle-income countries and underserved communities. Despite their exceptional durability, MHV recipients are at increased risk of thromboembolic complications. As such, the development of the next generation of MHVs must prioritize improved thromboresistance and aim for independence from anticoagulant therapy. However, innovation in MHV design faces several challenges: strict performance and biocompatibility requirements, limited understanding of the mechanisms underlying MHV thrombosis, and a lack of effective testing methodologies to assess how design variations impact both hemodynamic performance and thrombogenicity of MHVs. This paper reviews the emerging paradigms in MHV design, materials and surface modifications that may inspire the development of a new generation of MHVs for aortic valve replacement. We also discuss challenges and opportunities in developing experimental and numerical approaches to achieve a more comprehensive understanding of MHV flow features and the mechanisms of flow-induced blood clotting.

## 1 Introduction

**Figure 1:**
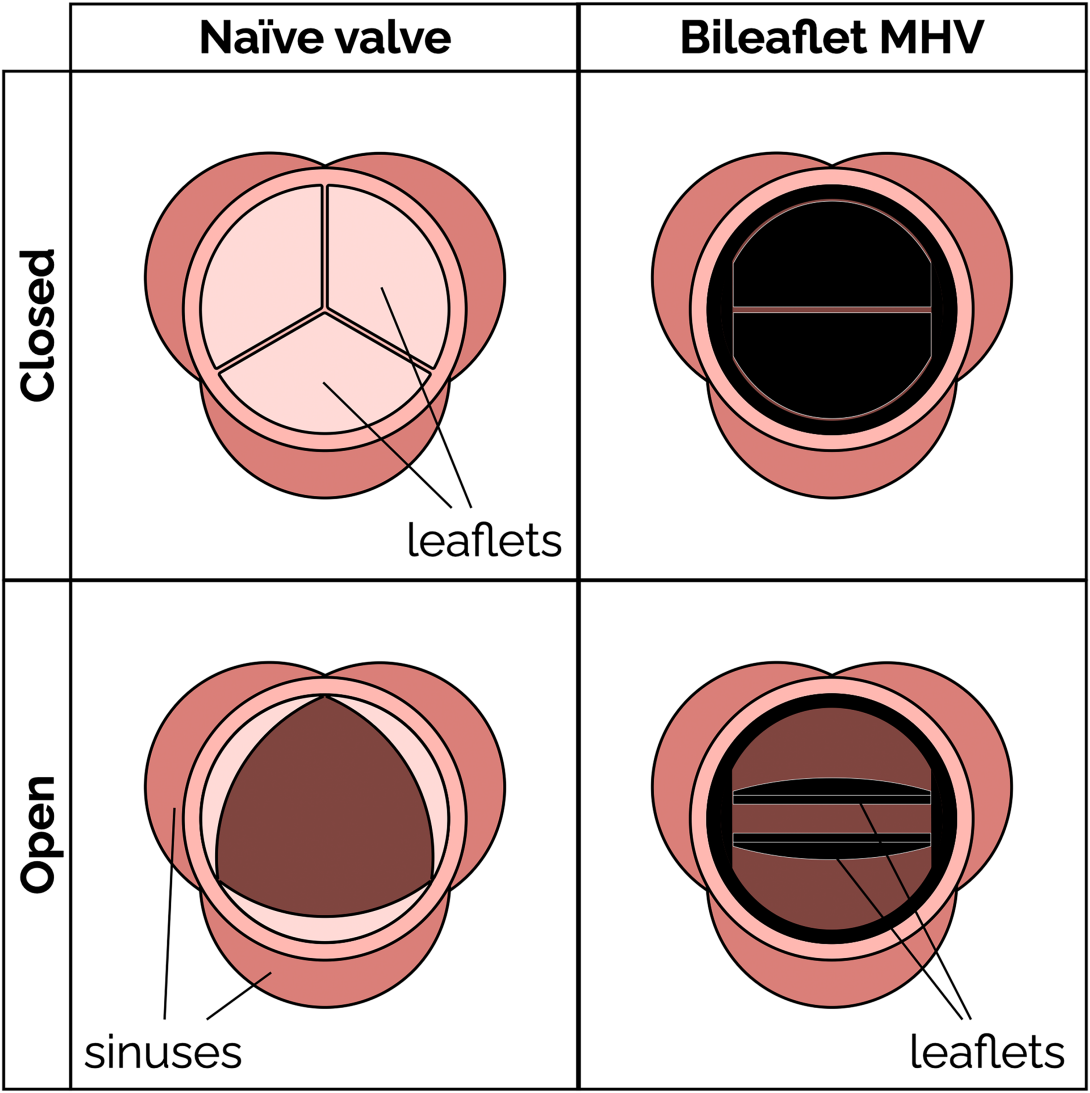
Schematic of a native aortic valve and a bileaflet mechanical heart valve in open and closed positions.

Aortic stenosis is a common heart valve disease in which an incomplete opening of the valve reduces flow of oxygenated blood from the heart to the aorta. Figure 1 shows the schematic of an aortic valve with three cusps, a common location for artificial heart valve implantation. Aortic stenosis is more prevalent in elderly people, and is diagnosed in about 12.4% of the US population older than 75 (1). Aortic valve replacement (AVR) is the treatment of choice for severe aortic stenosis, since treatment with pharmaceutical drugs has so far proven ineffective at curbing the progression of the disease (2).

Mechanical heart valves (MHV) (Figure 1) have been available for AVR since the 1960s. MHVs use pyrolytic carbon as the blood-contacting material (over a substrate in stainless steel, graphite or titanium alloys), because it provides good resistance to blood clotting (thromboresistance) (3, 4). A clinical and technological milestone was the introduction of the St. Jude Medical (SJM) bileaflet MHV in 1979. That valve offered exceptional durability combined with suitable hemodynamic performance and thromboresistance. Over the past 20 years, bileaflet MHVs have been replaced as the top choice for AVR by bioprosthetic heart valves (BHV), which can be implanted either surgically or over a catheter. BHV have superior hemodynamic performance and thromboresistance, and so they generally do not rely on lifelong anticoagulant therapy to prevent blood clotting complications (5).

MHV are still clinically preferred, particularly for some groups of patients, due to their superior durability. It is reported that 30% of implanted BHV's require replacement within 15 years, compared to only about 10% of MHVs (6–8). For that reason, current guidelines recommend implanting MHVs in people who are younger than 50 years and BHVs in patients with age more than 70 years (9). Recent studies have suggested an advantage of MHVs in patients between 50 and 70 years (10, 11). In addition to the durability issues, BHVs may cause clinical complications due to calcification (12, 13), and even require anticoagulant therapy in some cases (14, 15). MHVs are also the primary option for pediatric patients who are not eligible for the complex Ross procedure (16, 17), since BHVs have a history of poor outcomes in children and young adults (18, 19). At present, BHVs are more expensive than MHVs and difficult to access for lower-income patients (20). When BHVs fail, usually a transcatheter heart valve is deployed to prevent an open-heart surgery (21). Transcatheter valves have the same durability issues as surgical BHVs, and in addition they are susceptible to stenosis and prosthesis-patient mismatch (6, 22, 23).

Suboptimal thromboresistance of MHVs remains a major concern, especially for those groups where BHVs are not a viable option. Patients with an implanted MHV have an increasing risk of thromboembolism, due to the blood clotting induced by the prosthesis, as well as bleeding, which is a result of the anticoagulant therapy. Several studies have attempted to understand the mechanisms linking flow-induced clotting to specific factors contributing to MHV thrombosis, such as valve designs (24–30), size (31) and materials (32, 33). MHV design is one of the main culprits, because it is significantly different from the naïve aortic valve, thus producing non-physiological blood flow patterns (34–37), which may activate platelets and trigger the clotting cascade.

The goal of this paper is to provide a critical review of the current state and challenges of bileaflet MHVs in the aortic position. Understanding the design and research challenges that have slowed development and innovation for such an important medical device is an essential step for any scientist or engineer pursuing impactful research to pave the way for the next generation of anticoagulant-independent MHVs. In Section 2, we offer a primer on the main fluid-mechanic concepts underlying MHVs, including flow features and metrics relevant to blood clotting, as well as established design paradigms. In Section 3, we review a selection of emerging design concepts, materials and surface treatments that could improve hemodynamic performance and thromboresistance of future MHVs. In Section 4, we discuss experimental and numerical methods that have been developed to characterize MHV flow and flow-induced clotting, and the challenges that so far have prevented a comprehensive understanding of the mechanisms underlying MHV thrombosis. To strike a balance between the breadth and depth of our analysis, we chose to narrow the scope of this review to include primarily bileaflet MHVs implanted in the aortic position. Trileaflet MHVs are also briefly discussed in this review. Readers seeking to learn more can look elsewhere for detailed reviews of bioprosthetic valves (38, 39), transcatheter valves (23, 40, 41), and prosthetic mitral valves (13, 42). Polymeric prosthetic valves are an emerging area of research, and a comprehensive review of materials and recent developments has been published recently (43, 44). Finally, this review primarily focuses on the hemodynamics and thromboresistance aspects of MHV. Valve durability and wear are also important considerations, and are covered in detail in other reviews (45, 46).

## 2 Bileaflet mechanical heart valves

### 2.1 Bileaflet valve flow features

**Figure 2:**
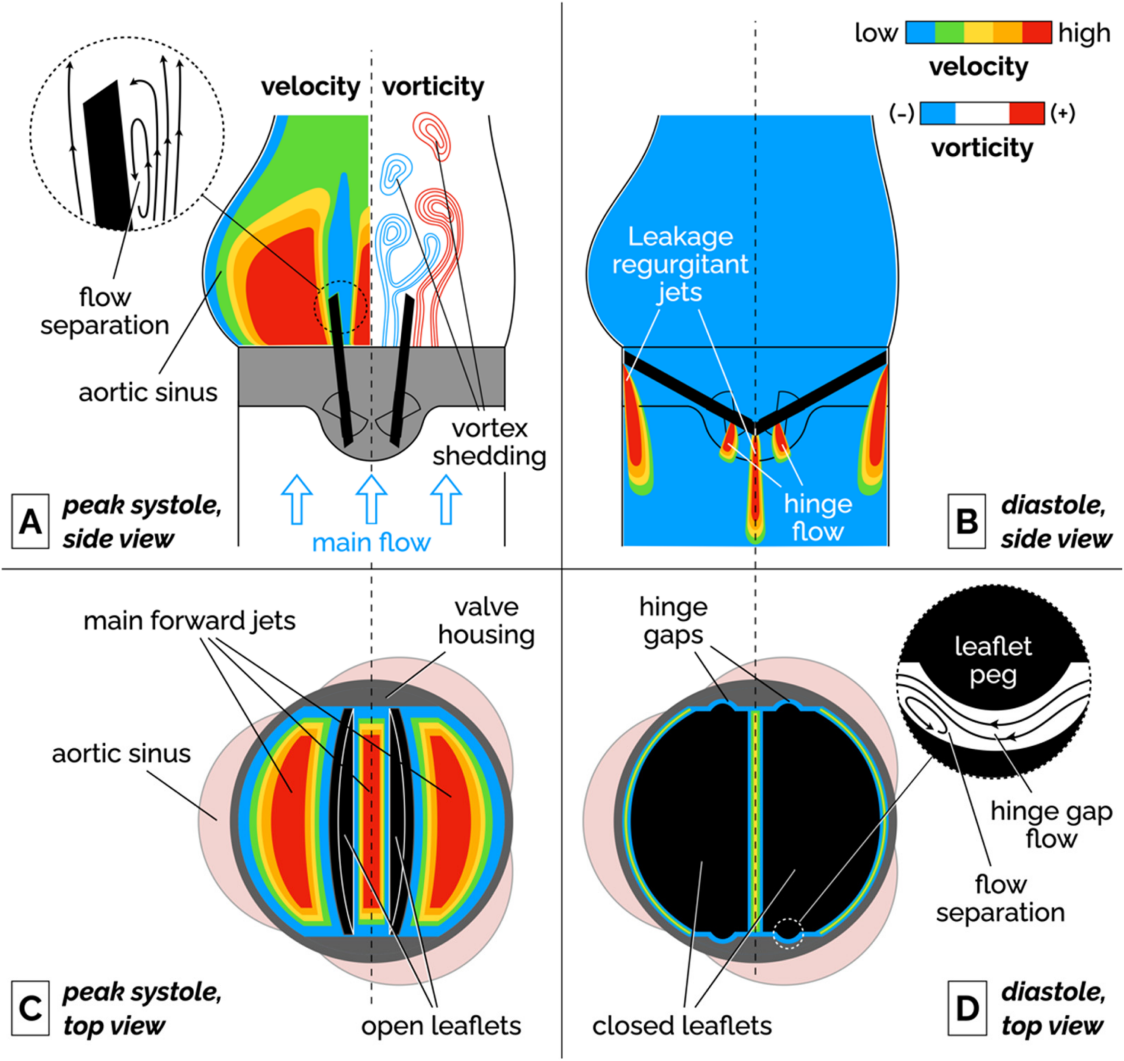
Schematic of the flow features of a bileaflet MHV. **(A)** Side view of the main forward flow at peak systole. Velocity and vorticity scales are qualitative. The vorticity map illustrates the shedding of pairs of counter-rotating vortices. The inset illustrates the flow separation region on the medial surface of the leaflet. **(B)** Side view of the regurgitant jets that develop at the edges of the leaflets and in the hinge gaps during diastole. **(C)** Top view of the valve during peak systolic flow. The fully open leaflets allow the development of three main jets. **(D)** Top view of the valve during diastole. The inset illustrates the streamlines and flow separation in the hinge gap between the leaflet peg and the butterfly-shaped recess in the valve housing. To keep the illustration simple, the hinge gap flow in the inset is seen from a different point of view than the top view image (right-to-left corresponds to the aorta-to-ventricle orientation).

Bileaflet MHVs in the aortic position exhibit distinct flow features compared to naïve aortic valves, primarily due to their rigid leaflet designs. During systole, blood flow creates three jets, through the central orifice and two lateral orifices on the sides of the fully open MHV, as illustrated in Figure 2A (side view) and 2C (top view). These jets exhibit high velocity and larger velocity gradients than naïve valves. In addition, depending on the shape and opening angle of the leaflets, wake flow can create non-physiological flow disturbances further downstream of the valve (35, 49). During diastole, MHVs are designed to have some degree of regurgitation (retrograde flow from the aorta back to the left ventricle). Regurgitation is a feature shared with naïve valves, but with some key differences. In bileaflet MHVs, it occurs as leakage flow through the valve hinges and is intended to wash out any blood that may be stagnating in the narrow hinge gaps. As a result, regurgitant flow in MHVs exhibits high-speed jets with abnormally elevated velocity gradients (much higher than in the forward three-jet flow during systole), which have been linked with mechanical shearing of platelets (35, 50, 51). Moreover, areas of flow recirculation can develop behind in the hinge regions, increasing the residence time of platelets and other blood cells, which may lead to thrombus formation. As an example, Dasi et al. reviewed the retrograde flow in the hinge gap of four commercially available valves (SJM standard, CarboMedics, Medtronic Parallel and Medtronic Advantage) and highlighted the recirculation zones that are trapped in this hinge recess (35). The Medtronic Parallel valve hinge design generated a highly convoluted flow compared to streamlined flows in the hinge gaps of the other valves. Eventually, the Medtronic Parallel MHV design was rejected after it showed severe blood clot formation near the valve hinge in clinical trials (35, 52). Figures 2B,D shows location of the hinge jets and flow recirculation during diastole (side and top view, respectively). In the remainder of this section, we will present some specific features of MHV flow that have been linked with potential thrombogenicity. Increasing the hinge gap size has been shown to improve washout of the stagnant flow at the hinges but causes the high velocities of the bulk flow to activate more platelets leading to increased thrombogenicity (50, 53).

It is important to point out that the flow features described above have been qualitatively and quantitatively characterized for fully-functioning, pristine MHVs, and so they are representative of ideal valve performance in the early stages after implantation. Leaflet malfunction, which may result in asymmetric leaflet motion, may produce substantially different levels of shear stress and flow stagnation or recirculation, thus accelerating MHV thrombosis (54, 55). Perhaps more importantly, progressive buildup of pannus and blood clots on the leaflets, hinges and housing of the valve may lead to radical changes in the hemodynamic performance of the MHV. Despite the clinical evidence that tissue and clot growth on the valve may occur even just months post implantation (56), *in-vitro* studies of non-pristine MHVs are a notable absence in the scientific literature.

#### 2.1.1 Flow separation, recirculation and stagnation

Fluid recirculation and stagnation resulting from flow through MHVs is primarily localized in or near the hinge regions, and occurs during diastole. Flow separation and low-shear zones have been observed (48), promoting blood stasis in these regions. These low-shear zones can activate platelets, significantly increasing the chance of thrombosis. The challenge in designing a MHV free of these issues lies in the nature of the flow's complex structure within the hinge, where small-scale turbulence and stagnation coexist. Proper hinge design can reduce thrombogenic potential but is difficult to optimize due to competing constraints on valve performance and durability.

#### 2.1.2 Vortex shedding

Flow separation has also been documented on the medial surface of the leaflet, which leads to adverse pressure gradients and vortex shedding in the wake of the valve. During flow acceleration at the beginning of systole, a pair of counter-rotating vortices develop downstream of the leaflet tip (57, 58). Eventually, these stationary vortices are disrupted by the onset of oscillations, which results in the alternating detaching of vortices from either side of the leaflet. This phenomenon, referred to as vortex shedding, is illustrated in the vorticity plot in Figure 2A. The wake of separated flow interacts with the flow jets creating low pressure zones of separated, pulsatile flow downstream of the leaflet. It has been suggested that platelets that had been activated in high-shear jets may get trapped in the vortices shed from the valve, potentially creating conditions favorable to platelet aggregation and clotting (54, 59, 60).

#### 2.1.3 Intermittent turbulent flow

As the flow decelerates and becomes more unstable during late systole, shed vortices are prone to secondary instabilities that transition into short-lived bursts of turbulent flow (61, 62). Because of the pulsatile nature of the flow, the Reynolds number drops from values above 6,000 at peak systole, to near zero during diastole. These conditions cannot sustain turbulence for the whole heart cycle. The turbulence generates increased viscous dissipation, contributing to higher energy losses. In particular, the interaction between the high-velocity jets and slower recirculating flows can drive flow instabilities, leading to intense localized turbulence, which contrasts with the smoother, less turbulent flow seen in naïve valves. Turbulent flow in MHVs encompasses multiple length scales from ∼25 mm through the valve orifice to ∼100 µm through the valve hinges (35, 63).

#### 2.1.4 Cavitation

Cavitation is the formation of vapor bubbles in a liquid usually at the interface of a moving solid body through the liquid (64, 65). In bileaflet MHVs, cavitation bubbles have been observed after the rapid closure of the valve at the end of systole. This large closing velocity of the leaflets leads to sudden pressure drops (28, 66). If liquid pressure locally drops below vapor pressure at body temperature, the formation of bubbles is possible. These vapor bubbles subsequently collapse, generating high-impact shock waves that can erode the valve material and damage surrounding blood cells (67, 68). Mohammadi et al. showed the acceleration of crack propagation in SJM valve leaflets due to water hammer pressure generated during cavitation (69). Due to rapid valve closure dynamics, the leaflets of Edwards Duromedics valves were subjected to cavitation leading to eventual fracture (70, 71). The distribution of cavitation bubbles depends on the valve geometry and kinematics (64, 68). Overall, the evidence available suggests that controlling cavitation may be important for both reducing the risk of thromboembolism and extending MHV durability (64).

### 2.2 Bileaflet valve flow metrics

Valve performance must be evaluated following ISO 5840-2, which outlines rigorous requirements for various hemodynamic and structural parameters for surgical valves (mechanical and bioprosthetic) (72). Key metrics include the effective orifice area (EOA) and total regurgitation fraction, which are crucial in assessing the functional efficiency and safety of MHVs. Total regurgitation fraction accounts for all backflow, including both closing volume and leakage volume. While not strictly required by ISO 5840, transvalvular pressure gradients and shear stresses are commonly reported to characterize flow hemodynamic performance and thrombogenic potential of MHVs.

#### 2.2.1 Transvalvular pressure gradient (TPG)

The transvalvular pressure gradient (TPG) is the pressure drop between the ventricular and aortic side of a MHV and represents a measure of the loss of potential energy across the valve. Ideally, TPG should be very low so that the valve does not considerably impede the blood flow; however, ISO 5840-2 does not include a specific requirement for the acceptable range of TPG in MHVs. Compared to a native aortic valve, the rigid material of MHVs create a smaller flow area that increases the pressure drop. Large TPG values are highly undesirable because it means increasing the pressure in the left ventricle during systole, which if sustained may lead to conditions like ventricular hypertrophy and even heart failure. Table 1 reports the TPGs for seven types of commercial MHVs.

**Table 1:** Typical values of flow parameters for popular MHVs.

Valve type	Size (mm)	TPG (mmHg)	Peak RSS in a cardiac cycle (Pa)	Time averaged peak VSS (Pa)	EOA (cm^2^)	Regurgitant volume (ml/beat)	References
Medtronic parallel	25–27	22 (6)	200–800	–	2.73–3.07	8.4–8.9	(36, 73, 74)
ATS	18	15.3 (2.9)		–	1.44	7.7 (1.1)	(75, 76)
Carbomedics	21	15.1 (2.1)		–	1.66, 1.45	3.4, 7.5 (0.5)	(36, 75, 77)
	23		690	–	2.28	6.51	(36, 77)
Sorin Bicarbon	19	10.4 (2.2)	551	–	1.59	4.3 (0.9)	(73, 75, 78)
SJM Standard	19		207	–	1.21	6.8	(36, 78)
	23		160–200	–	2.24	8.3	(36, 79)
	27		125	60	4.09	10.8	(36, 80, 81)
SJM Regent	19	8.7 (0.8)	–	48–60	1.78	8.6 (1.6)	(75)
	23	4.75 (0.05)	72, 133, 260	5–15	2.36	6.3–10.3	(37, 77, 82)
On-X	19	14.5 (4.3)	–	53–60	1.8	6.5 (1.3)	(75)
	23	4.15 (0.09)	95, 58.4	106	2.61	6–7.8	(82, 83)
Apex^a^	25	–	–	–	–	–	(84)

TPG, transvalvular pressure gradient; RSS, Reynolds’ shear stress; VSS, viscous shear stress; EOA, effective orifice area.

^a^ The Apex valve is a newly proposed design. Experiments are under progress.

#### 2.2.2 Effective orifice area (EOA)

During forward flow of blood through MHVs, the extent of valve opening is measured by the Effective Orifice Area (EOA) as defined by Equation 1:

(1) $$\mathrm{EOA}({\mathrm{cm}}^{2})=\frac{Q_{rms}(mL/s)}{51.6\;\sqrt{\frac{\Delta P(mmHg)}{\rho (g/cm^{3})}}}$$

where *Q_rms_* is the root mean square forward/systolic flow rate, *ΔP* is the mean pressure across the valve during ejection, and *ρ* is the density of the test fluid. EOA should be estimated from measurements of pressure and flow in a pulse duplicator simulating realistic physiological conditions. It is important to use the units indicated in Equation 1, since the unit conversion factors are already baked into the “51.6” coefficient (85). MHVs should be designed to make EOA as large as possible, to minimize the pressure drop across the valve for efficient valve function. It is related to valve shape, size and closing mechanism of leaflets. If we consider for example a MHV with nominal size of 27 mm, ISO 5840-2 requires a minimum EOA of 1.70 cm^2^.

#### 2.2.3 Total regurgitation volume

Total regurgitation volume is the total volume of blood that backflows through the valve during one heart cycle, due to retrograde flow during valve closing and reverse leakage flow through the hinges. Total regurgitation is typically reported as a percentage of the stroke volume. MHV design should seek to minimize the regurgitant volume to maximize the heart's efficiency by maintaining the correct cardiac output of the body (35, 36). For a MHV with nominal size of 27 mm, ISO 5840-2 requires a maximum total regurgitation fraction of 15%. In Table 1, total regurgitation volumes for some commercial valves have been reported as observed in few experiments.

#### 2.2.4 Shear stresses

Shear stresses are important metrics of the hemodynamic performance and thrombogenic potential of MHVs. They have the dimensions of force per unit area (or energy per unit volume), and are related to velocity gradients (velocity per unit length) and fluid viscosity:

(2) $$\tau =\mu \dot{\gamma }$$

In Equation 2, $\dot{\gamma }$ is a generic shear rate, while *µ* is the apparent viscosity of the fluid. For Newtonian behavior, there is a linear relationship between shear stress *τ* and shear rate $\dot{\gamma }$.

Historically, two distinct metrics of shear stress have been reported, sometimes interchangeably even though they are conceptually different. One kind of shear stress can be obtained as in Equation 2, thus representing a measure of the local, instantaneous viscous forces in the fluid (viscous shear stress, or VSS). Alternatively, shear stress can be calculated from the fluctuating components of fluid velocity in turbulent flows. This second type of stress is referred to as Reynolds shear stress (RSS) and represents a measure of the turbulent fluctuations in the flow (82, 86). Both viscous shear stresses (VSS) and RSS have been linked to hemodynamic efficiency and elevated VSS and RSS have been found to contribute to thrombogenicity of MHVs (37, 80, 87).

Hemodynamic efficiency of MHVs decreases when excessive shear stresses develop in the flow. These high shear forces lead to greater viscous energy dissipation, which negatively affects both TPG and EOA. Elevated shear stresses in MHV flow can develop in high-speed jets as well as intermittent bursts of turbulent flow in the wake of the valve. These flow irregularities create inefficiencies, increasing the workload on the heart and affecting overall valve performance.

Shear stresses exceeding normal physiological levels are responsible for platelet activation (an important precursor of blood clotting) or even platelet lysis. The precise mechanisms that relate abnormal shear stresses to clotting are still unclear and have been a major focus of MHV research for decades. It appears that important mechanical factors should include the intensity and duration of platelet exposure to elevated VSS, as well as the occurrence of turbulence.

Experimental measurements of VSS done by various groups with SJM Regent heart valves show a high variability as seen in Table 2. Klusak et al. (80) showed that although mean VSS is much lower (60 Pa) than the threshold for hemolysis (400–800 Pa (35, 36) the *instantaneous* peak VSS (120 Pa) is above the shear stress criterion for platelet activation (10–100 Pa (35). A similar result was obtained from an earlier numerical study (94). A precise threshold called Hellum's criterion was defined as the product of shear stress and the time duration. Platelets will activate if this value is above 3.5 Pa (35). All these critical values were obtained from *in vitro* measurements.

**Table 2:** Design trends found in MHVs in commercial use or in development.

Valve type	State	Opening angle	Housing shape	Housing material	Hinge	Leaflet shape	Leaflet material	Refs
Medtronic Parallel	Discontinued from clinical use.	60^o^	Cylindrical	Graphite +20% Tungsten	Sudden expansion and contraction in circular hinge	Straight	Graphite + PyC + 20% Tungsten	(35, 88)
ATS	Commercially available	85^o^	Cylindrical	Graphite + 20% Tungsten	Open Pivot Hinge	Straight	Graphite + PyC + 20% Tungsten	(75, 88, 89)
Carbomedics	Commercially available	78^o^	Cylindrical	PyC	Male portion of butterfly hinge on the leaflet.	Straight	Graphite coated with PyC	(90)
Sorin bicarbon	Commercially available	70^o^	Cylindrical	Titanium	Chamfered butterfly hinge recess so that leaflets can roll instead of slide.	Curved	PyC	(75, 91)
SJM standard + SJM Regent	Commercially available	85^o^	Cylindrical	Graphite	Male portion of the butterfly hinge on the leaflet.	Straight	Graphite with PyC	(75, 92)
On-X	Commercially available	90^o^	Cylindrical	Graphite	Male portion of butterfly hinge on the leaflet. Smaller recess than SJM.	Straight	Graphite + 10% tungsten	(82, 93)
Apex bileaflet MHV	Newly proposed design	89^o^	Saddle shaped	PEEK	Female portion of the hinge is on the leaflets.	Curved	PEEK	(84)

PyC, pyrolytic carbon; PEEK, polyetheretherketone.

Peak RSS values in different types of MHV are shown in Table 2, although there is some discrepancy in reported measurements of RSS. Earlier studies reported peak RSS during the valve closure mid-diastolic phase (260 Pa for 23 mm SJM Regent valve) (95) while recent work shows peak RSS is observed during peak systole for the same valve (72 Pa) (82).

Interestingly, exceedingly low levels of shear stress are also detrimental, since they may signal the occurrence of regions of flow recirculation or stagnation, where conditions may be favorable to the aggregation (coagulation) of previously activated platelets (96, 97).

Understanding the role of shear stresses in platelet activation and controlling them through MHV design improvements is crucial to achieving independence from anticoagulants. Current MHV designs have been refined to minimize shear stresses, while avoiding development of flow circulation and stagnation regions, as will be discussed in the next section, but clearly there is still much work to do to reduce shear stresses to levels comparable to naïve and bioprosthetic valves.

### 2.3 Current bileaflet MHV designs

The design of bileaflet MHVs has been stagnant, with one of the most popular models, the St. Jude Medical (SJM) Regent valve being only an incremental refinement of the original model introduced in 1979. Since then, bileaflet MHVs have essentially supplanted all the other mechanical designs (namely ball-and-cage and tilting disc valves). At the same time, their adoption rate has decreased due to the hemodynamically superior bioprosthetic and—more recently—transcatheter tissue valves.

Table 2 summarizes the main design features of selected bileaflet MHVs. The criteria for inclusion in the table were the availability of detailed information on valve design and performance from the open literature and the historical or potential significance of their design. Apart from the Medtronic Parallel valve (discontinued in the 1990s due to unacceptably high levels of clotting in the hinges) and Apex valve (still in development), all the valves in Table 2 are still being implanted in new patients. We did not include any ball-and-cage and tilting disc valves, although they may still be encountered in long-term follow-up patients.

**Figure 3:**
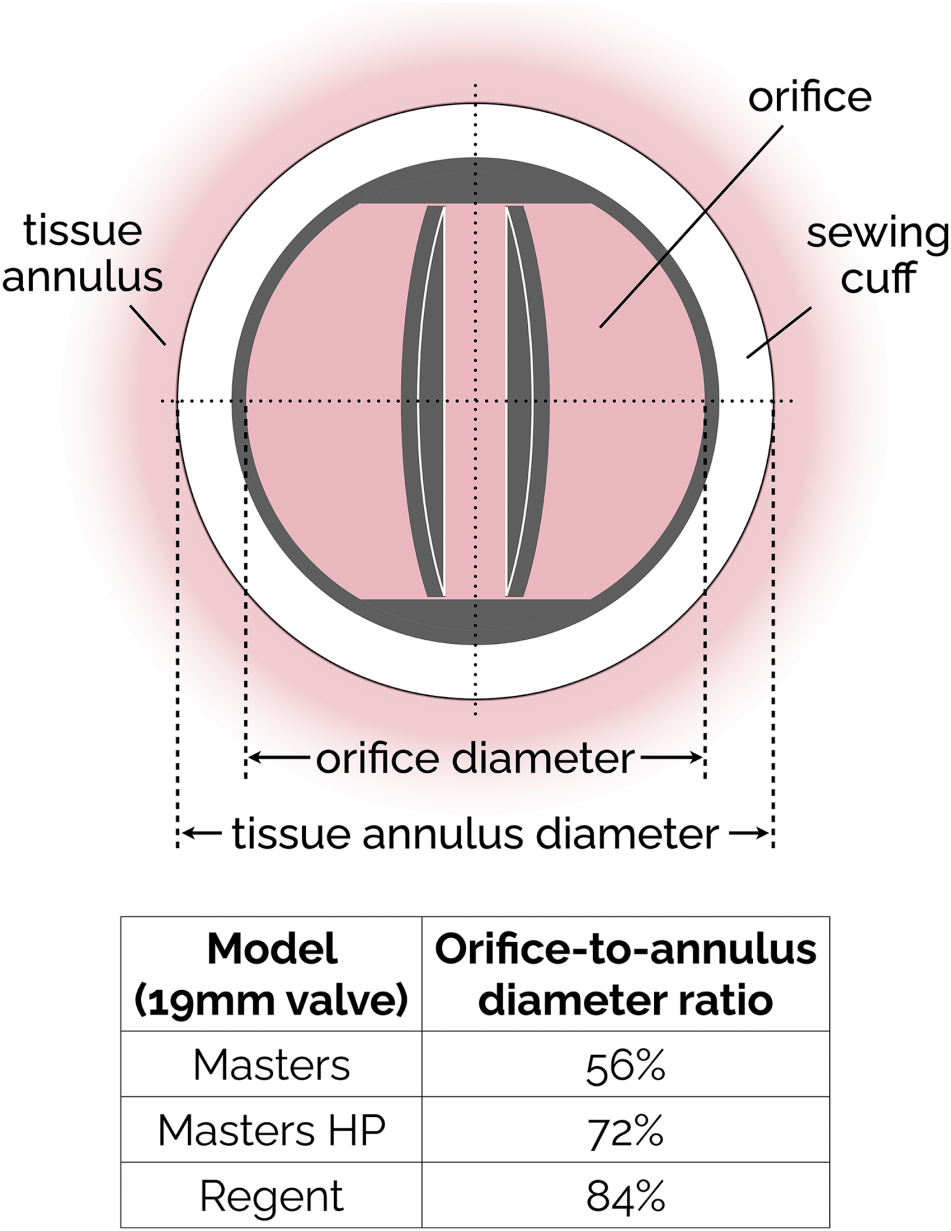
Simplified sketch of the SJM bileaflet valve. In subsequent iterations (from the original Masters design to Masters HP and finally to Regent), the sewing cuff has been shrunk and mover from intra-annular to supra-annular position, resulting in an increase of the orifice-to-annulus diameter ratio. The ratio values reported in the figure are specific to the 19 mm version of the SJM valve.

The basic design of bileaflet MHVs features two leaflets (usually D-shaped) hinged to a one-piece housing fitted with an outer suture ring for implantation in the aortic root (98). The SJM Regent valve, a popular version of this design, is illustrated in Figure 3 as an example. It is an incremental evolution than earlier versions of the SJM bileaflet valve, which are now referred to as Masters and Masters HP valve. The main design change from Standard/Masters to Masters HP to Regent was the repositioning of cuff and sewing ring, which helped increase the orifice-to-annulus ratio from 56% (Standard valve) to 72% (Masters HP valve) to 84% (Regent valve), resulting in improved EOA and TPG (99). The hinges follow a peg-and-recess design, with the recess usually located in pockets within the housing, and they allow for the leaflets to open to a maximum angle (relative to the annulus plane) between 60° and 90°. Most MHVs are designed for adult patients, with nominal dimensions between 19 and 31 mm. The only MHV approved for pediatric patients is the 15-mm SJM Masters HP valve.

The Sorin Bicarbon MHVs featured curved leaflets, which was a major departure from the flat design of the SJM valves. In addition, the hinges of the Sorin valves were designed to roll instead of slide (91). Overall, the performance of the two valves was similar, with the Sorin exhibiting higher EOA and smaller regurgitation (73, 75, 78).

The hinge designs of most MHVs include a butterfly shaped recess with the male configuration (Table 2) where the leaflet moves within the hinge recess. The On-X has a smaller recess than the SJM valves. The hinges with the female configuration where the protrusions from the hinge attach to the leaflets offer superior hemodynamics as shown by the newly proposed Apex/iValve design (84, 100).

For most MHVs, the surfaces in contact with blood are made from pyrolytic carbon, a synthetic material developed in the 1950s that exhibits low friction and wear and remarkably good thromboresistance. In most cases, pyrolytic carbon is used as a coating to the more durable materials used for the bulk of leaflets and housing, such as graphite and titanium alloys. Exceptions to these materials are the recent On-X valve, which uses tungsten, and the still-in-development Apex valve, whose prototypes were made either from aluminum or PEEK. PEEK is a rigid polymeric material that has recently been investigated for its good durability and thromboresistance (4, 101).

## 3 Recent trends in MHV design

The incremental evolution of the popular SJM bileaflet MHV design is illustrative of the limited innovation over the past 50 years. To the best of our understanding, that is partly due to the research challenges that have hampered a better understanding of the mechanisms of flow-induced blood clotting in MHVs (as discussed section 4), and partly due to the rise of alternative bioprosthetic valves (first surgical, and then transcatheter ones), which for a time were expected to completely supplant MHVs for aortic valve replacement. However, MHVs are still the preferred option for specific populations (20, 53, 102), and recent efforts to innovate design, materials and surface treatment of MHVs have revived the hope that they may achieve independence from anticoagulants.

**Figure 4:**
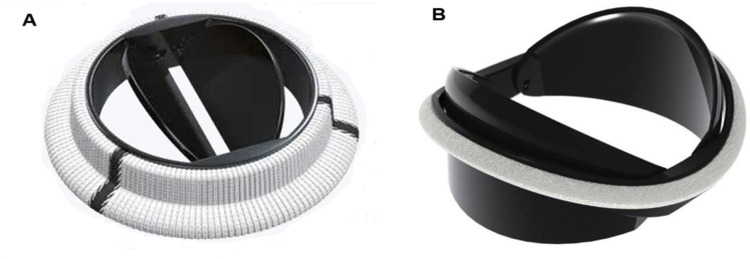
Bileaflet valves in open position **(A)** On-X valve design. Reproduced with permission from “0n-X@ Aortic Heart Valve” by Artivion, Inc. Used with the Permission of Artivion, Inc. **(B)** Newly proposed iValve design. Adapted with permission from “Rendered isometric views of the iValve are shown in the A) closed and B) open positions. Both the housing and leaflets are identifiable” by Dylan Goode, Lawrence Scotten, Rolland Siegel and Hadi Mohammadi, licensed under CC BY-NC 4.0.

Hinge design, number and curvature of the leaflets and closing dynamics have been the focus of recent research. The On-X aortic bileaflet MHV (Figure 4A) was approved by the US FDA in the early 2000s and marketed with an emphasis on the reduced need for anticoagulation therapy (103). In many ways, the On-X valve represented a departure from the traditional MHV design. The length-to-diameter ratio is closer to a naïve aortic valve. A leaflet opening angle of 90° was the largest yet (Table 2), and combined with a tapered housing inlet, an actuated pivot and leaflet contact at two points, it improved valve hemodynamics and reduced thrombogenicity (93) compared to existing MHVs. One major drawback of the On-X valve is the occurrence of leaflet migration (104–106), which has rarely been observed in other types of MHV.

More recently, a new valve design was proposed with the promise to significantly reduce thrombus formation due to stagnant blood in the hinge region (84). An earlier version of this design, dubbed Apex, (Table 2) featured saddle shaped housing, female portion of butterfly hinges on the leaflet instead of the male portion, and a one-point contact between the leaflets and the housing (84). Subsequently, Mohamadi et al. introduced the iValve MHV as an evolution of their Apex valve (100). The iValve housing has a saddle shape that pushes blood flow towards the hinge ensuring proper washout. The hinge design is different from the Apex design, since the butterfly shaped appendage has been replaced by placing a pie shaped appendage on the housing with a C-shaped open socket on the leaflets.

The curved leaflets of the iValve (Figure 4B) effectively eliminate the lateral jets of traditional bileaflet valves, resulting in a single central jet (107). Based on the limited results reported so far, this major design change may improve hemodynamics and increase the effective orifice area (100). For instance, the closing time and volume of the iValve are improved compared with SJM Regent and ON-X valves (107). The backflow velocity was lower with no fluctuations. The TPG of the iValve is also better compared to the SJM and ON-X valves. While still in development, the Apex/iValve design is an example of innovation that may pave the way for significantly better MHVs.

### 3.1 Trileaflet MHVs

The concept of a trileaflet mechanical valve is not particularly novel (24–26, 29, 30, 108), but some recent studies have rekindled the interest in this design (109–111). Trileaflet MHVs feature a main central jet, plus three relatively smaller side jets, which reportedly results in smaller velocity gradients compared to bileaflet MHVs (28, 112). The closing mechanism of a trileaflet MHV also appears to create smaller velocity gradients and prevent cavitation, as the swirling vortices during decelerating phase of forward flow initiate closing earlier than bileaflet MHVs (113).

Trileaflet MHVs have been investigated extensively *in vitro* (27, 109, 110, 113), in silico (28, 114–116) and in animal studies (24–26, 30, 108, 111, 117). Although preclinical trials showed promising results in terms of blood compatibility, no trileaflet MHV has been approved yet for clinical use. Several research groups tested the hemodynamic performance of the Lapeyre-Triflo MHV, manufactured by Triflo Medical Switzerland. The leaflet opening angle of the Lapeyre-Triflo valve is 90 degrees, which matches that of the On-X bileaflet valve and is higher than most commercial MHVs. Reportedly, the increased opening angle reduces disturbance in the forward flow by increasing the EOA for the same valve size (53). In preclinical studies, the thrombogenic potential of the Lapeyre-Triflo valve in calves was comparable to bileaflet MHVs (108, 111). A recent *in vivo* implantation of a novel 21-mm Lapeyre-Triflo valve in sheep showed promising preliminary results when monitored over 1 year post surgery (117). TPGs were low, regurgitation was minimal, no hemolysis was detected and thrombogenicity was low even without anticoagulation therapy.

### 3.2 Surface materials, coatings and treatment

Pyrolytic carbon has been the blood-contacting material of choice for MHVs for decades, and is used in many MHVs due to its superior hemocompatibility and favorable mechanical properties (118). For instance, valves made using titanium alloy with a pyrolytic carbon coating provide excellent structural stability as well as decreased thrombogenicity (91). Unfortunately, even the level of thromboresistance provided by pyrolytic carbon is not sufficient to prevent blood clotting. Table 2 lists the materials used for housing, leaflets and coating for few commercially available bileaflet MHVs.

Blood-contacting materials alternative to pyrolytic carbon have been explored. Tetrahedral carbon and ultra nano crystalline diamond have shown the potential to improve wear resistance and chemical resistance (4). Ceramics commonly used for orthopedic and dental implants may provide benefits as a coating material for MHVs, due to their wear resistance and stability (53). Without compromising structural integrity, their use may help reduce leaflet thickness, with a concomitant potential to increase EOA. Carbon-ceramic materials have also been tested, and it has been suggested that they may achieve biocompatibility equivalent to pyrolytic carbon, even with substantially thinner coatings (119, 120).

Other studies have proposed coatings to be added to the pyrolytic carbon surfaces, to further improve thromboresistance (121). Heparin coatings can provide additional protection to clotting, but only in the short term, until the drug is completely eluted into the bloodstream (122–124). Attempts to modify the surface chemical composition by applying a dense titanium oxide coating on pyrolytic carbon (125) or by implanting nitrogen ions to the surface (126) have shown only marginal benefits. Research on the addition of superhydrophobic coatings to pyrolytic carbon has shown some potential. Superhydrophobic coatings trap thin layers of air on valves and thereby decrease the contact area between solid and liquid (127). The small contact areas decrease blood cell adhesion and friction to the pyrolytic carbon surface (32, 127–130). Unfortunately, experimental work has shown mixed results with regard to the efficacy of these coating materials in reducing the likelihood of platelet damage and activation. Bark et al. reported that for a contact angle of 160°, a superhydrophobic spray on a pyrolytic carbon surface eliminated adhesion of platelets and leukocytes. The performance index (PI) of the coated valve improved by 2.5% compared to an uncoated valve (32). Hatoum et al. further investigated the hemodynamic parameters like RSS and instantaneous VSS for a 3D-printed MHV with superhydrophobic coating. The flow metrics that are correlated with platelet damage did not change showing that superhydrophobicity does not improve valve hemodynamics (131).

Finally, various kinds of surface treatment have been proposed to modify its topology to make it more thromboresistant (4). Laser ablation is a cost-effective, environment-friendly, precise and quick technique to create superhydrophobic surfaces on different materials, as the surface roughness can be switched from nanoscale to microscale conveniently (132–134). Laser etching of the pyrolytic carbon of SJM valves (127) (135) produced an increase in the contact angles, which may be conducive to improved hemodynamics, via reduced flow resistance, and thromboresistance. Both hemolysis rate and flow pressure drop reduced further when the superhydrophobic surfaces were laser processed on PyC. The non-equilibrium surface tension in a gradient hydrophobic surface drives droplet movement that can potentially reduce turbulence, mitigating thrombus formation (136).

### 3.3 Vortex generators

**Figure 5:**
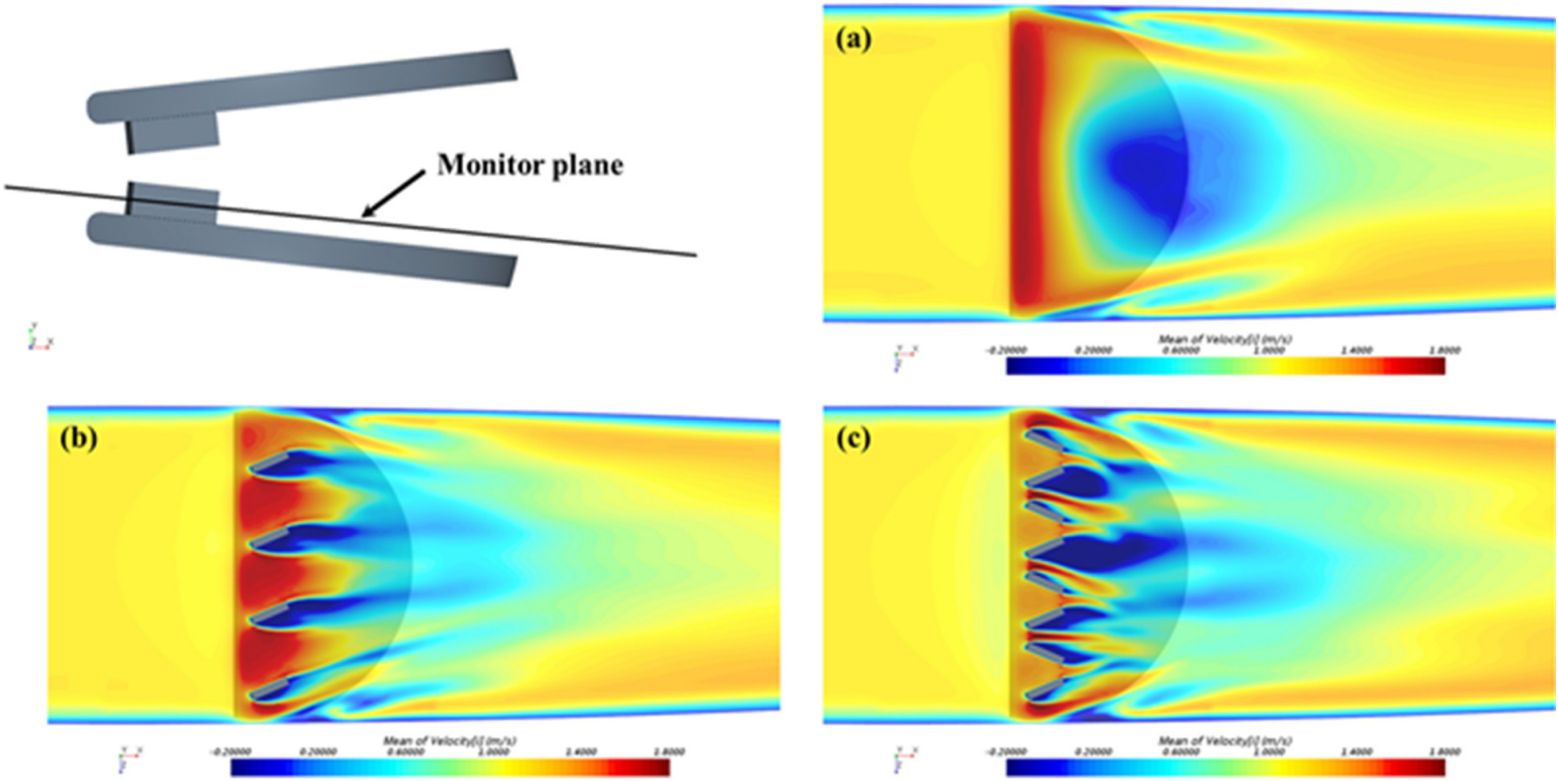
Time-averaged streamwise velocity contours in the monitor plane: **(a)** control: without VGs; **(b)** with co-rotating VGs; and **(c)** with counter-rotating VGs. Reprinted with permission from “Time-averaged streamwise velocity contours in the monitor plane: (a) control: without VGs; (b) with co-rotating VGs; and (c) with counter-rotating VGs” by Zhenyu Wang, Lakshmi Prasad Dasi and Hoda Hatoum, licensed under CC-BY.

The surface of MHV can be altered by the addition of passive flow control elements, referred to as vortex generators (VGs). VGs are placed on the MHV leaflet surface (137) to delay flow separation and reduce turbulent shear stresses (33). It has been reported that VGs can mitigate the thrombogenic potential of the turbulent jet that develops in the narrowing gap between the two leaflets during closing (138). One downside is the drag penalty associated with VGs (33, 139). PIV and CFD studies have investigated the effect of shape, height, spacing and configuration of VGs, to maximize the hemodynamic benefits while minimizing the excess drag (33, 58, 137, 139, 140). Hatoum et al. showed that a co-rotating equally spaced configuration of rectangular shaped VGs on a 23-mm 3D-printed MHV (Figure 5) offered the optimal performance with improved EOA, minimum TPG and turbulence during pulsatile flow conditions in a mock circulation loop (33, 139). Computational studies by the same author showed that a co-rotating VG configuration can reduce blood cell damage by 4.7% in comparison to a valve without VGs (139). The same study showed that counter-rotating configurations proved to be detrimental as it increased the blood damage by 3.7%. Recent computational studies by Salleh et al. investigated different configurations of triangular shaped VGs on rigid valve leaflets under steady state conditions (141). The authors reported that the inclusion of VGs may have a negative impact on the EOA. In a follow-up study, they used Large Eddy Simulation (LES) to compare the effect of using triangular VGs on an axisymmetric aorta and an anatomic aorta. The anatomic design was found to be more susceptible to thrombosis due to higher peak velocity (2.03 m/s) and WSS (69 Pa) during peak flow phase compared to the axisymmetric one (140).

The experimental studies with VGs conducted so far have not been able to characterize the local flow between VGs, due to limited spatial resolution. Computational studies are able to resolve those flow features, but the computational cost of time- and space-resolved FSI simulations including non-Newtonian effects of blood near the VGs represents a challenge. In addition, more research is needed to investigate additional combinations of the VG shapes and configurations with moving MHV leaflets, which may also help clarify the effect of VGs on the EOA (141).

## 4 Research challenges

### 4.1 Flow characterization techniques

An important aspect of the research on MHVs is the detailed characterization of the flow in the proximity of the valve, especially because MHV flow exhibits abnormal features that are distinct from naïve valve flow. The flow dynamics in the proximity of the valve are crucial to understanding the hemodynamic performance of MHVs, as well as the potential complications that may arise post-implantation, such as thrombosis, hemolysis, or structural valve deterioration. First, MHV flow characterization can be used to identify abnormal features during systole, when abnormal flow patterns such as turbulent jets, flow separation, or regions of high shear stress may develop, and diastole, when regions of stagnation, vortex formation, and reverse flow can emerge. Additionally, MHV flow characterization allows one to directly or indirectly estimate the valve performance metrics discussed in Section 2. These metrics are typically characterized in controlled laboratory environments using mock circulation loops (MCL), also known as pulse duplicators, capable of replicating physiological flow near a test MHV. To assess valve performance according to ISO 5840-2, most MCLs are equipped with pressure and flow sensors, which can capture pointwise or area-averaged data with sufficiently high temporal resolution.

To understand the underlying mechanisms of MHV thrombosis and test the effect of design changes, a more detailed characterization of the flow aims at measuring the velocity field within the flow. Eulerian velocity maps with sufficiently high spatial and temporal resolution can be used to estimate important features, such as local and convective accelerations, shear rates, vorticity, Lagrangian trajectories and coherent structures (47, 55, 142). In addition, if assumptions are made on the mechanical response of the fluid to shearing (e.g., if Newtonian flow behavior is assumed), one can estimate viscous shear stresses and Reynolds shear stresses, as well as the time-dependent mechanical loading experienced by a platelet along a Lagrangian trajectories (143, 144). These estimates have been used in combination with blood damage index (BDI) models to quantify the total damage potentially experienced by a platelet during a single pass through the MHV. There is some evidence that accumulated damage (even below lethal thresholds) may lead to platelet activation and coagulation (145, 146).

**Figure 6:**
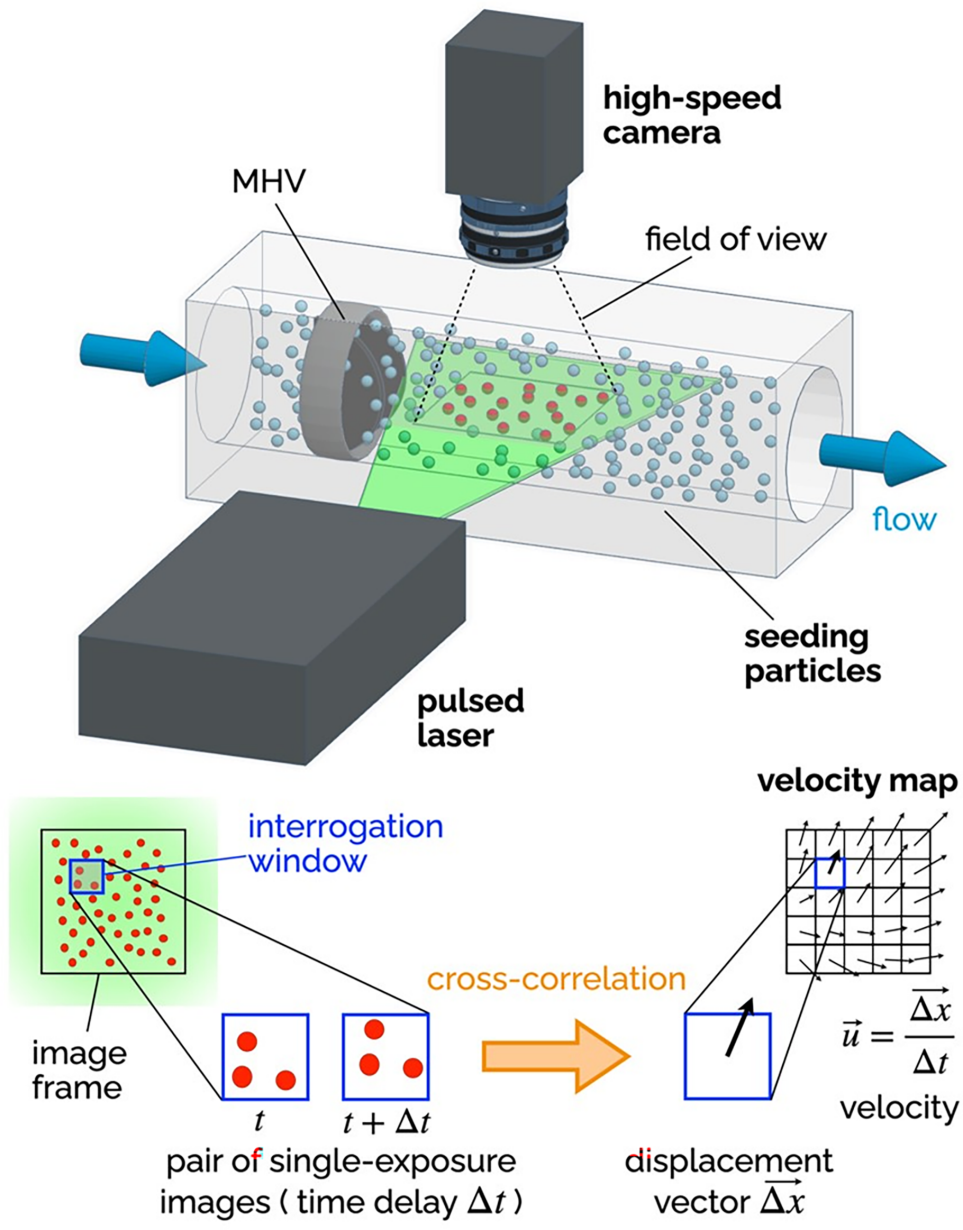
Principles of 2D-2C PIV. A high-speed camera captures images of the seeding particles in the plane illuminated by a thin laser sheet (pulsed). Each image is divided into a grid of interrogation windows. Pairs of single-exposure images (delayed by *Δ*t) are analyzed using cross-correlation algorithms to determine the particle displacement *Δ*x (in the plane of the laser sheet) for each interrogation window. The result is a vector map of the two components of the fluid velocity in the plane of the laser sheet. The delay *Δ*t affects the temporal resolution of the velocity maps, while the distance between the center points of the interrogation window affects the spatial resolution.

Since fluid velocity data are so critical, researchers have used experimental and numerical techniques to measure it. Particle Image Velocimetry (PIV) includes several techniques, all based on the same foundational principles, illustrated in Figure 6. The flow is seeded with neutrally-buoyant scattering or fluorescent particles, a slice or volume of the flow is illuminated (typically with a laser source), images of the illuminated particles are collected in pairs, each pair of images is divided into small interrogation windows and analyzed with cross-correlation algorithms to estimate the particle displacement vector *Δ*x for each interrogation window. The calculated vector *Δ*x/*Δ*t (where *Δ*t is the temporal separation between the two images) represents the velocity of the flow at a specific position in the flow (associated with an interrogation window). PIV is commonly used for the flow characterization of cardiovascular devices, as it allows for time- and space-resolved velocity data. Depending on the specific PIV technique, it is possible to measure 2 or 3 components of fluid velocity either in a planar or a volumetric domain of the flow.

Planar two-component (2D-2C) PIV has been extensively used to non-invasively investigate the MHV flow features during *in vitro* experiments. The recommendations for effectively using PIV to calculate important metrics like residence time, flow velocity and shear stress have been compiled by Raghav et al. (147). 2D-2C PIV was used to quantify velocity fields near valve leaflets under both steady (29, 148) and pulsatile flow conditions (55, 137, 149, 150). Experimental studies with PIV have shed light on the characteristics and relative strength of the three jet-flow observed during systole, when the valve is fully open (55), as well as the role of flow instability developing in the decelerating flow during late systole, which may trigger short burst of turbulent flow in the ascending aorta (47). 2D-2C PIV has also helped characterize the high-speed jets that develop in the narrow gaps of the valve hinges during diastole, when the valve is fully closed (80, 151). All valve designs include some level of hinge clearance, for washout purposes, but hinge flow during diastole is prone to high shear rates, which may lead to platelet activation or even damage.

Because 2D-2C PIV can only measure two components of the velocity in one plane, most studies have focused exclusively on the in-plane velocity in the midplane perpendicular to the rotation axis of the leaflets. Several studies have used more advanced PIV configurations than 2D-2C PIV to overcome the inability to resolve the out-of-plane velocity components (152). Stereoscopic PIV (planar domain, three components of velocity) has allowed for three-dimensional reconstruction of turbulent kinetic energy and vortical structures downstream of MHVs, which for instance has demonstrated the importance of valve orientation when the valve is used in the mitral position (153). Hinge jets with high three-dimensional velocity and turbulence were accurately captured with mechanical and bioprosthetic heart valves in multiple studies exhibiting steady (154) as well as pulsatile flows (152, 155, 156). The limitation of stereo-PIV technique is that the volume of interest is reconstructed from different 2D measurement planes. This requires complex setups to move the PIV system, time-consuming calibration, and post-processing techniques. The introduction of tomographic (157) and holographic PIV (volumetric domain, three components of velocity) enabled capturing all three velocity components simultaneously over a volume of interest. 3D PIV studies investigated the flow topology behind bioprosthetic valves (158) and trileaflet MHVs (159). A recent tomographic PIV study with a MHV in the mitral position analyzed the complete evolution of the vortical structures during a heart cycle and reported lower turbulent kinetic energy levels during mid-diastole and systole compared to tilting-disc valves (160).

Ongoing developments in PIV data processing algorithms, along with the introduction of higher-speed higher-resolution cameras, will lead to even more detailed characterizations of MHV flow dynamics, against which numerical models and clinical data can be compared. However, PIV suffers from some major limitations which are particularly relevant to MHV studies. First and foremost, PIV requires a transparent fluid, which prevents the use of whole blood in this kind of study. Moreover, the need for adequate optical access to the fluid domain under investigation makes it difficult to measure flow near the valve (e.g., hinge flow). To overcome this limitation, researchers must use optically clear models of the aortic root, and in some cases even clear models of the MHV. Glass, acrylic, polycarbonate and silicone elastomers have been used in combination with either water or blood analogues, typically a 40:60 mixture of water and glycerol to replicate blood viscosity (xanthan gum can be added to better model the non-Newtonian behavior of blood). Unfortunately, every combination of solid and fluid listed above results in a mismatch of their refractive index, which creates optical distortion and reduces the accuracy of velocity measurements from PIV (161–163). Another limitation of PIV is that, even with the most advanced cameras and laser sources currently available, resolution may fall short of resolving all the relevant temporal and spatial scales of MHV flow (37, 47).

While PIV is the most common *in vitro* flow measurement technique for MHV, some earlier studies adopted Laser Doppler Velocimetry (LDV), which is a well-established velocity measurement technique that allows to collect high-quality data from a single point in the fluid domain (51, 164). LDV has some advantages over PIV, namely higher accuracy and temporal resolution, no need for flow seeding, and lower sensitivity to refractive index mismatch (165). However, PIV is superior because of its ability to visualize entire flow fields simultaneously, which makes it possible to capture flow patterns and structures.

As briefly mentioned above, *in vitro* flow characterization studies commonly rely on test fluids exhibiting Newtonian behavior (water-glycerol mixtures, or just water). These fluids at best replicate the viscosity of whole blood (about 3.5 cP at high shear rates, where the stress-shear rate curve becomes approximately linear), but they fail to replicate both the nature of whole blood (a concentrated suspension of cells) and its non-Newtonian behavior. While it is generally accepted that the rheological behavior of blood in the aorta is largely Newtonian, as shear rates are above 100 s^−1^, there is some evidence that studies with Newtonian fluids may underestimate the extent of blood cell damage associated with MHV flow (166).

Because of their reliance on laser illumination, PIV and LDV can only be used in *in vitro* studies. These techniques require precise optical access to the flow field, making them unsuitable for direct use in living organisms, where such access is typically obstructed by biological tissues. However, in the context of this review it is worth mentioning two techniques that can be employed both *in vitro* and *in vivo*, providing crucial insights into the hemodynamic performance of MHVs in clinical and research settings: phase-contrast magnetic resonance imaging (PC-MRI), and echocardiography particle image velocimetry (Echo-PIV). PC-MRI is a non-invasive imaging technique that can be used to visualize and quantify blood flow, providing time-resolved three-component velocity data (also known as 4D flow MRI), making it suitable for capturing complex flow patterns and turbulence downstream of MHVs (167, 168). Echo-PIV is an emerging technique that combines the principles of traditional PIV with ultrasound imaging to measure flow velocities *in vivo* and *in vitro* (169). In Echo-PIV, microbubbles or contrast agents are introduced into the bloodstream to act as tracer particles, and their motion is tracked using high-frame-rate ultrasound imaging. By analyzing the movement of these particles, Echo-PIV can generate detailed velocity fields similar to those produced by PIV, but in a non-invasive, *in vivo* context. Echo-PIV is still a relatively new technique, but it holds great promise for bridging the gap between *in vitro* and *in vivo* studies of valve performance. In addition, it has the potential to be used for monitoring patients with implanted MHV (170).

### 4.2 Numerical approaches

Computational fluid dynamics (CFD) have been extensively used to simulate the three-dimensional hemodynamics of MHVs to obtain reproducible, faster, cost-effective insight into the flow physics (50, 171–173). Some of the simpler models simulate MHV flow with fixed leaflets combined with either steady or pulsatile flow conditions, to investigate the triple jet MHV flow dynamics when the leaflets are fully open (36, 171, 174). More refined approaches may prescribe leaflet motion, as derived from experiments, to better simulate MHV flow features with moving leaflets (55, 174).

More comprehensive approaches employ coupled fluid-structure interaction (FSI) algorithms, where leaflet motion is not prescribed but calculated by the model simultaneously with the pulsatile flow. A review by Sotiropulos et al. describes the detailed findings from FSI simulations that investigates the vorticity dynamics, asymmetrical leaflet motion and the effect of implanted valve orientation on MHV hemodynamics (95). Recent advancements in the coupling methods of the fluid and structural domains and developments in individual fluid-structure solvers have enabled numerical simulations of patient-specific hemodynamics across heart valves (175). Abu Bakar et al. (176) validated a FSI model from PIV data to compare velocity and vorticity in a MHV flow domain. Fully coupled two-way FSI studies are ideal for investigating MHV dynamics where fluid and structural domains are solved in parallel (177). Mutual interactions between valve leaflets were accounted for through implicit coupling methods (178, 179). These studies are frequently done using high performance computing (HPC) clusters depending on the model complexity and variability of model parameters.

Grid based methods with fixed grid, moving grid or a combination of both are conventionally used in these solvers. The k-*ω* shear stress transport (SST) model numerically solves the flow equations and calculates turbulent kinetic energy, turbulent dissipation in addition to velocity and shear stress fields (180, 181).

In a different approach, termed the particle approach, the fluid phase is modeled by the lattice Boltzmann method (LBM) as a distribution of fictitious fluid particles. Results from this approach can be used for Lagrangian tracking of platelet activation or observing the influence of hematocrit on MHV hemodynamics (94, 182, 183). The standard bounce back (SBB) method added to the LBM improves numerical stability for retrieving accurate MHV flow parameters while the external boundary force (EBF) technique of the LBM considers platelets as points with no volume and can help accurately predict platelet activation and blood damage (175).

Overall, alternative computational methods like smooth particle hydrodynamics have shown promise in investigating native heart valves (184, 185) that can extend to study of MHVs. The recent advent of physics-informed neural networks (PINNs) can enable future studies of patient specific MHV hemodynamics and integrate them with multi-modality data to provide a comprehensive model.

PINNs can personalize in-silico models obtained from 4D-MRI cardiac images based on the anatomy and the microdetails of the vasculature (186). The strength of this method is its flexibility to define all flow variables as approximate functions of space and time that can approximately match the boundary conditions and measurement data. PINN studies enable wall shear stress calculation with sparse data where inflow and outflow boundary conditions were not well defined (187). Recent work employed PINN algorithms to investigate flow through a transcatheter aortic valve (188), abdominal aorta (189) and the left ventricle (186). Future implementation of PINN in predicting non-invasive hemodynamic measurements can accelerate patient specific treatment and diagnosis.

### 4.3 Blood clotting studies

MHV flow characterization in MCLs with transparent blood analogues is useful for comparative studies, as it can provide indirect evidence of thrombogenicity such as shear stresses, residence times and blood damage indices. However, this kind of study alone is not sufficient to investigate all the factors involved in the complex mechanisms of platelet activation and clotting. To establish a link between abnormal MHV flow features and clot formation, whole blood should be used (5, 14, 186).

Early studies of flow-induced blood clotting focused on exposing whole blood to controlled flow conditions. Those studies demonstrated that prolonged exposure of cells to abnormally high-shear stress is responsible for both hemolysis (187, 188), platelet lysis and activation (189). Experiments in steady mean flow have shown that hemolysis levels are significantly higher in turbulent flow than in laminar flow at the same mean shear stress (190). Importantly, those early studies helped identify reference threshold values of viscous shear stress for platelet lysis and activation, and contributed to the idea of sublethal damage accumulation, which may activate platelets over multiple passings through the MHV, eventually leading to coagulation. These findings have been included into empirical models that predict platelet damage from shear stress and residence time data (143, 145, 191). It is important to point out that early studies of flow-induced blood clotting relied on simplified flow systems, such as cone-and-plate rheometers, and thus could not replicate realistic MHV flow.

Some studies have attempted to characterize MHV flow-induced clotting using enzyme-activated milk as the test fluid. This approach is based on some analogies between the mechanisms of clotting of enzyme-activated milk and whole blood (110, 192, 193). Even fewer studies have exposed MHVs to whole blood. One example is the small-volume, biocompatible pulse duplicator developed at Georgia Tech (194). The system was designed to require only 150 ml of whole blood, which could be obtained from a single donor. Results included tests with a 23-mm St. Jude Medical Regent valve in the aortic position. The same system was used for both MHV flow characterization, with PIV and a transparent blood analogue, and coagulation studies, with freshly drawn human blood. Another example is the THIA (Thrombosis Tester Helmholtz Institute), a platform that has gone through several iterations. The latest generation (THIA 3) is a pulse duplicator suitable for evaluating initial clot formation in MHV under realistic anatomic and hemodynamic conditions (195). The ability of THIA 3 to compare the thrombogenic potential of MHVs was validated using a 23-mm St. Jude Medical Regent valve and a test valve (made from polycarbonate urethane) with geometrical and surface features designed to make it highly thrombogenic.

### 4.4 The need for novel experimental approaches

There are only a few *in vitro* studies where mechanical heart valves are tested with whole blood under realistic hemodynamic conditions. The main reason is the incompatibility of the commonly used laser-based techniques (PIV, LDV) with opaque fluids such as whole blood. In addition, it is challenging to design a pulse duplicator where the thrombogenic effect of MHV flow can be isolated from other potential sources of blood clotting, such as other foreign surfaces in the loop or mechanical loading caused by other parts such as pumps or resistance valves. One promising solution has been recently proposed by Devos et al. (102). Their MarioHeart system is based on a 155-ml circular loop, housing a MHV, which is accelerated alternately clockwise and counterclockwise. In one phase, the relative motion between the MHV and blood opens the valve, simulating systole; in the other phase, the flow closes the valve, mimicking diastole. This design makes it possible to replicate MHV flow in whole blood while eliminating surface and mechanical elements that could confound the analysis and interpretation of clotting results. The systems just described (102, 194, 195) represent promising solutions to the challenge of *in vitro* testing of MHVs, and hopefully will inspire more investigators to shed light on the link between MHV detailed flow features and blood clotting.

## 5 Future directions

The next generation of MHVs needs to deliver on the promise of mitigating thrombogenic risk for the many patients who still receive this kind of valve. This ambitious goal requires innovation in valve design, materials and surface modifications. The review of recent research involving MHVs underscores some of the recent progress made in the last decade, for instance trileaflet MHVs, valve designs with a single central jet like the Apex/iValve concept, or the potential of PEEK as an alternative to pyrolytic carbon. These ideas show that MHVs superior to the traditional bileaflet MHV design are possible. Current developments in 3D printing and rapid prototyping technologies can be used to fabricate and test multiple design variations of bileaflet and trileaflet MHVs. However, prototyping valves coated with pyrolytic carbon (for direct comparison with commercial MHVs in thrombogenicity tests) remains a challenge for many academic laboratories.

The future of MHV thrombosis research also encompasses innovation in testing, across *in vitro*, in-silico and preclinical platforms. This will allow more accurate, comprehensive and reproducible solutions to investigate flow-induced clotting mechanisms and the effect of novel design paradigms on MHV thromboresistance.

While traditional laser-based techniques such as PIV and LDV provide valuable insights into valve flow dynamics, their limitations with opaque fluids like blood have hindered testing with whole blood. Innovation in *in vitro* testing means overcoming those limitations and achieving a tighter integration of flow characterization and thrombogenicity testing systems. Currently, the closest integration attainable *in vitro* is a pulse duplicator that can be run with a clear blood analogue, for detailed flow characterization, and whole blood to study the onset and growth of blood clots. One such example is the MarioHeart system, which promises the ability to simulate realistic conditions with whole blood as the test fluid, although it remains to be proven that it can isolate the thrombogenic potential of MHVs from other confounding factors present in the pulse duplicator.

Whereas a dual-fluid platform suitable for either a clear fluid or whole blood may prove challenging, the second best option would be a twin system with separate clear-fluid MCL and whole-blood thrombogenicity tester. This is a viable approach, provided that the two testers produce matching flow profiles, so the detailed flow data from PIV or LDV can be combined with the clotting data from the whole blood tests.

An alternative approach to experimental research on MHV thrombosis may rely on non-laser-based flow characterization techniques, such as PC-MRI and echo PIV. Because neither method requires optical access to the flow, they can be used with whole blood, allowing to collect detailed flow and clotting data simultaneously. While PC-MRI is only available in few research facilities and the resolution of echo PIV is still limited compared to laser-based PIV, the potential of these techniques for *in vitro*, preclinical and clinical studies of MHV thrombosis cannot be overstated.

CFD will play an increasingly pivotal role, because it allows for the efficient and systematic exploration of design variations, to identify the most promising features to be subsequently tested in more focused *in vitro* studies. To accomplish that, there is a need to increase the resolution of multiscale FSI models, while keeping the computational cost reasonably low. Looking further ahead, future numerical approaches will have to go beyond pure fluid dynamics, by integrating mechanochemical models of flow-induced blood clotting (196).
